# Genetically Modified Soybean Detection Using a Biosensor Electrode with a Self-Assembled Monolayer of Gold Nanoparticles

**DOI:** 10.3390/bios12040207

**Published:** 2022-03-30

**Authors:** Cheng-Chi Chou, Ying-Ting Lin, Iren Kuznetsova, Gou-Jen Wang

**Affiliations:** 1 Department of Mechanical Engineering, National Chung-Hsing University, Taichung 40227, Taiwan; chou70911357@gmail.com; 2 Program in Tissue Engineering and Regenerative Medicine, National Chung-Hsing University, Taichung 40227, Taiwan; jeff0213@gmail.com; 3 Kotelnikov Institute of Radio Engineering and Electronics, Russian Academy of Science, 125009 Moscow, Russia; kuziren@yandex.ru; 4 Graduate Institute of Biomedical Engineering, National Chung-Hsing University, Taichung 40227, Taiwan; 5 Regenerative Medicine and Cell Therapy Research Center, Kaohsiung Medical University, Kaohsiung 80708, Taiwan

**Keywords:** monolayer of gold nanoparticle sensing electrode, self-assembled monolayer, electrochemical impedance spectroscopy, genetically modified soybeans, label-free detection

## Abstract

In this study, we proposed a genosensor that can qualitatively and quantitatively detect genetically modified soybeans using a simple electrode with evenly distributed single layer gold nanoparticles. The DNA sensing electrode is made by sputtering a gold film on the substrate, and then sequentially depositing 1,6-hexanedithiol and gold nanoparticles with sulfur groups on the substrate. Then, the complementary to the CaMV 35S promoter (P35S) was used as the capture probe. The target DNA directly extracted from the genetically modified soybeans rather than the synthesized DNA segments was used to construct the detection standard curve. The experimental results showed that our genosensor could directly detect genetically modified genes extracted from soybeans. We obtained two percentage calibration curves. The calibration curve corresponding to the lower percentage range (1–6%) exhibits a sensitivity of 2.36 Ω/% with R^2^ = 0.9983, while the calibration curve corresponding to the higher percentage range (6–40%) possesses a sensitivity of 0.1 Ω/% with R^2^ = 0.9928. The limit of detection would be 1%. The recovery rates for the 4% and 5.7% GMS DNA were measured to be 104.1% and 102.49% with RSD at 6.24% and 2.54%. The gold nanoparticle sensing electrode developed in this research is suitable for qualitative and quantitative detection of genetically modified soybeans and can be further applied to the detection of other genetically modified crops in the future.

## 1. Introduction

Traditionally, selective breeding has been used as the standard method of crop improvement. With global extreme weather and the continuous increase in population, selective breeding has been unable to effectively solve the problem of food shortage and other issues. Therefore, scientists began to use genetic engineering technology to improve crops and make them resistant to diseases and insect pests, herbicides, extreme weather, etc., in order to increase food production and nutritional value [1].

Gene transfer technology selects those crops with desirable characteristics. Genetically modified crops (GMC) are engineered by inserting a gene into the DNA of the plant to produce an effective insecticide called Bacillus thuringiensis (Bt). GMCs have been widely planted to increase yield per unit area, reduce pesticide use, and increase food nutrition. The International Service for the Acquisition of Agribiotech Applications (ISAAA) had reported that 185.1 hectares of GMCs were cultivated worldwide in 2016 [2]. GMCs are mainly planted in the USA, Canada, Argentina and China [3]. The U.S. Center for Food Safety has reported that 85% of corn produced in the U.S. is genetically modified. Soybeans, cotton, corn and rape are the four main GMCs. Among them, soybeans are the largest [4], accounting for 82% of the total soybean planting area. Although GMCs are increasingly gaining acceptance in the USA, Argentina, Canada and China, there is still strong consumer rejection in European countries due to concerns that genetically modified foods have a potential impact on human health [5,6,7]. The major issue is the unknown risk due to the transplantation of Bt genes into crops. According to the report by the Center for Food Safety, Bt corn can induce an allergic response due to the original DNA of the corn having been modified. The International Regulations and Codex guidelines stipulate the bio-safety requirements of GMCs. If the genetically modified organism (GMO) content surpasses the standards of a recommended threshold, GMCs and their products are compulsorily labeled. Therefore, making use of sensitive detection methods, both at the DNA and protein levels for the timely detection of GMCs is urgently needed [8,9].

At present, the detection of genetically modified crops focus on screening specific genetic traits (qualitative) and the proportion of genetically modified ingredients in food (quantitative). Qualitative tests are mainly carried out by polymerase chain reaction (PCR) [8,10], PCR-based detecting methods [11,12,13,14], enzyme-linked immunosorbent assays (ELISA) [15,16] or rapid tests (rapid screening reagents, test paper) [17], while quantitative tests are mainly carried out by RT-PCR [18,19]. The processes include sample preparation, DNA extraction and purification, DNA amplification and GM target detection. Although these techniques are considered highly convenient and productive, they are time-consuming, relatively expensive, and always require highly trained personnel [20]. However, detection can be easily performed using sensitive DNA biosensors, such as electrochemical, optical, and piezoelectric biosensors [21]. DNA biosensors have been considered a highly feasible DNA detection technology that would gradually replace current PCR approaches and provide portable, fast and ultrasensitive GMC detection.

Among those reported DNA biosensor schemes, electrochemical DNA biosensors have been the most popular due to their advantages of relatively low-cost, high sensitivity, high selectivity and versatility of detection principles [22]. Many electrochemical DNA biosensing schemes have been reported recently based on electrochemical impedance spectroscopy (EIS) [23,24], differential pulse voltammetry (DPV) [25], square wave voltammetry (SWV) [26,27], and chronoamperometry (CA) with enzymatic amplification [28,29] principals.

Wang et al. [23] reported a label-free electrochemical impedimetric DNA biosensor for the detection of the cauliflower mosaic virus 35S (CaMV 35S) in a DM soybean with a linear detection range of 1 × 10^−16^ M–5 × 10^−10^ M and a detection limit of 3.3 × 10^−17^ M. Sun et al. [25] proposed an electrochemical DNA sensor based on a partially reduced graphene oxide-modified carbon ionic liquid electrode for the sensitive detection of target ssDNA sequences related to the transgenic soybean A2704-12 sequence. A linear detection range for PCR products of transgenic soybeans was measured to be from 1.0 × 10^−12^ to 1.0 × 10^−6^ mol/L, with a detection limit of 2.9 × 10^−13^ mol/L. Manzanares-Palenzuela et al. [30] proposed magnetoassays that integrated electrochemical detection with end-point PCR for the quantitative analysis of genetically modified soybeans with the GTS-40-3-2 event (also known as Roundup Ready soybeans). Electrochemical measurement was conducted on screen-printed carbon electrodes. A linear range of 2–250 pM for event-specific and taxon-specific targets, with detection limits of 650 fM (160 amol) and 190 fM (50 amol), respectively, was obtained. Aghili et al. [26] proposed an electrochemical nanobiosensor based on an exfoliated graphene oxide and gold nano-urchin modified screen-printed carbon electrode for quantitative detection of genetically modified organisms. A linear range of 40.0–1100 fM with a limit of detection of 13.0 fM was obtained. However, in these existing electrochemical DNA biosensors, the testing samples were usually synthetic DNA, or a reference material with PCR, or a real sample with PCR. A PCR-free electrochemical DNA biosensor for GMC detection using samples directly extracted from real crops is desired.

Due to the rapid development of gene-editing technology, many GMCs have been constructed in a programmed form and carry the cauliflower mosaic virus (CaMV) gene sequence from the soil-borne bacterium Agrobacterium tumefaciens. According to statistics, more than 80% of GMCs were constructed using the CaMV 35S promoter (P35S) and the NOS terminator (TNOS) [31]. If the P35S can be discerned, crops with this GM trait can be effectively detected. Therefore, this research focuses on GM soybeans containing the P35S DNA sequence to develop a low-cost, simple operation and to develop a rapid detection sensing electrode and its corresponding electrochemical detection method [32]. It is also hoped that the results of this research can be applied to the qualitative and quantitative testing for specific genetic traits of other GMCs in the future.

## 2. Materials and Methods

### 2.1. Materials and Reagents

1,6-hexanedithiol (1,6-HDT), 6-mercapto-1-hexanol (MCH), potassium chloride, potassium phosphate monobasic, and sodium chloride were purchased from Sigma-Aldrich (Burlington, MA, USA). Sodium phosphate dibasic was purchased from J. T. Baker (Phillipsburg, NJ, USA). Potassium ferricyanide and potassium ferrocyanide were purchased from SHOWA (Antarctica, Japan). Distilled deionized water (>18 MΩ) was obtained from ELGA LabWater (High Wycombe, UK).

### 2.2. Experimental Process

**Scheme 1:**
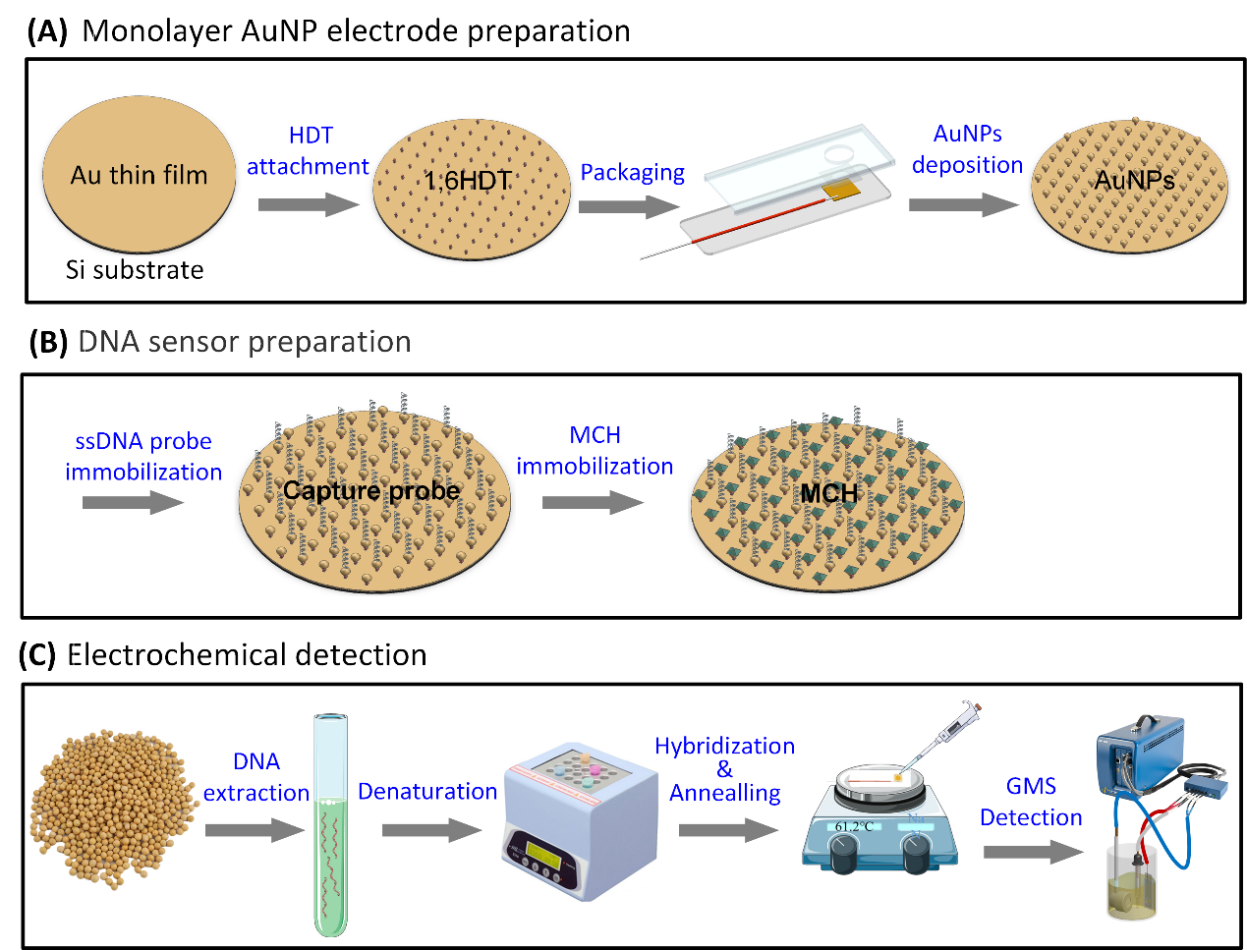
The experimental framework of this research. (**A**) Monolayer array of the AuNPs electrode preparation, (**B**) DNA sensor preparation, and (**C**) electrochemical detection.

Scheme 1 describes the experimental framework of this research, including (A) monolayer array gold nanoparticles (AuNPs) electrode preparation, (B) DNA sensor preparation, and (C) electrochemical detection.

#### 2.2.1. Monolayer AuNPs Electrode Preparation 

An Au thin film on a 1.5 cm × 1.5 cm silicon substrate was sputtered using a sputter coater (Model 108 Auto, Cressington, Watford, UK) with a current of 30 mA, a sputtering pressure of 0.08 mbar, and a time of 135 s. The reason for using silicon substrate was that the annealing temperature after hybridization was 61.2 °C and commonly used polymer materials cannot withstand this temperature. Next, the sample was dipped into a 50 mM 1,6-HDT solution for 18 h to enable one thio-end of the 1,6-HDT to attach to the thin gold layer [33]. Cleaning was then repeated with 75% alcohol three times to remove excess HDT, followed by the application of high-pressure nitrogen to remove excess residual alcohol on the surface. After packaging, a 10 wt% colloidal AuNP solution was dropped onto the dithiol-modified electrode to obtain the monolayer AuNPs electrode (Scheme 1A).

#### 2.2.2. DNA Sensor Preparation 

A gene fragment that is related to the CaMV 35S promoter (P35S) complementary thiolated capture ssDNA probe was used to detect genetically modified soybean (GMS). The sequence of the probe, 5′ GCT CCT ACA AAT GCC ATC AT 3′, contains 20 bp of nucleotide (Genomics, New Taipei City, Taiwan). The 5′ end of the probe was modified with a thiol group. After dripping 33 μL of the 0.5 μM probe solution onto the electrode and overnight incubation, an ssDNA probe was immobilized on the electrode via a thiol-Au interaction. Next, 33 μL of the 2.5 mM 6-mercapto-1-hexanol (MCH) solution was added to the probe-ssDNA immobilized electrode and incubated for 4 min to block the non-immobilized area (Scheme 1B).

#### 2.2.3. Electrochemical Detection

(1) Target dsDNA preparation

The double-stranded target DNAs from genetically modified soybean (GMS) and organic soybean (OS) were extracted using the GeneJET Plant Genomic DNA Purification Kit (ThermoFisher Scientific, Waltham, MA, USA). The 1X annealing buffer (ThermoFisher Scientific, Waltham, MA, USA) was used to dilute the extracted GMS DNA, and the extracted OS DNA to the target dsDNA solutions of 1%, 2%, 3%, 5%, 6%, 10%, 20%, 40% and 80%. The target dsDNA solutions were denatured at 95 °C for 10 min to form single-strand DNA. The diluted target DNA solution (33 μL) was then dripped onto the sensing electrode, incubated on an electromagnetic heating stirrer at 61 °C for 10 min, and then cooled at room temperature for 5 min, followed by washing with DD-water.

(2) Detection of genetically modified soybean

A three-electrode SP-150 potentiostat (Bio-Logic, Seyssinet-Pariset, France) was implemented to distinguish the GM and OS soybeans through electrochemical impedance spectroscopy (EIS). The working electrode, counter electrode and reference electrode were, respectively, the as-fabricated sensing device, the Pt film and the Ag/AgCl. A mixture of 5 mM K_3_[Fe(CN)_6_], 5 mM K_4_[Fe(CN)_6_] and 0.1 M KCl in phosphate buffer saline (PBS) (pH = 7.0) was used as the buffer solution. The applied DC power and AC power were 0 V and 10 mV, respectively. The scanning AC frequency was between 0.01 Hz and 100 kHz (Scheme 1C).

### 2.3. Target dsDNA PCR Amplification and Gel Electrophoresis Analysis

In order to confirm whether the extracted target dsDNA contained the Rbcl soybean gene fragment and the CaMV 35S promoter (P35S) genetic modification gene fragment, the P35S forward primer (5′GCT CCT ACA AAT GCC ATC AT3′), P35S reverse primer (5′GAT AGT GGG ATT GTG CGT CA 3′), Rbcl forward primer (5′ATG TCA CCA CAA ACA GAG ACT AAA GC3′), Rbcl reverse primer (5′GTA AAA TCA AGT CCA CCR CG3′) and other specific primers were used to amplify target dsDNA to 245 molecules. Gel electrophoresis analysis was then conducted.

### 2.4. The Quantification of dsDNA

The Invitrogen Qubit 4 fluorometer (Invitrogen, Waltham, MA, USA) and the Qubit™ 1X dsDNA HS assay kit (Invitrogen, Waltham, MA, USA) were used to confirm the target dsDNA concentration.

## 3. Results and Discussion

### 3.1. Characterization of the Monolayer AuNPs Electrode

**Figure 1:**
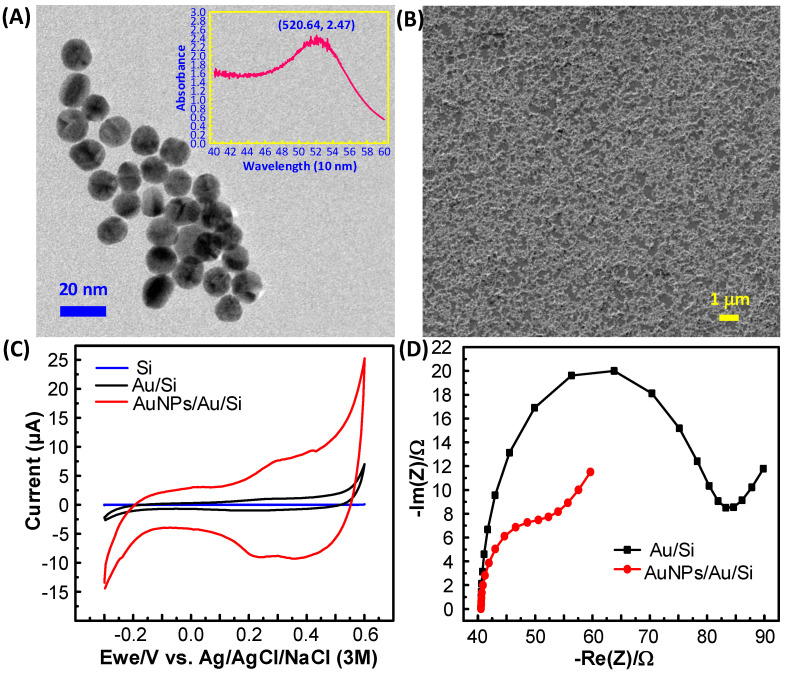
Electrode characterization. (**A**) TEM image of the colloidal AuNPs used for forming the monolayer AuNPs electrode on the silicon substrate, (**B**) SEM image of the monolayer AuNPs electrode on the silicon substrate, (**C**) CV curves of the fabricated electrodes, and (**D**) Nyquist plots of the fabricated electrodes.

A monolayer of AuNPs was formed on the silicon substrate through a self-assembled monolayer process. Figure 1A shows the transmission electron microscopic (TEM) (JEM-2010, JEOL, Tokyo, Japan) image of the colloidal AuNPs used to form the monolayer on the surface of the Au thin film-coated silicon substrate using HDT. Using ImageJ, the average size of AuNPs was estimated at ~13.5 nm. The UV-vis absorbance spectrum shown in the inset indicates that the AuNPs had an absorption peak at 520 nm, corresponding to an AuNP’s size of 13 nm. Figure 1B shows the scanning electron microscopic (SEM) (JSM-7800F, JEOL, Tokyo, Japan) image of the monolayer AuNP on the silicon substrate. Figure 1C,D depicts the cyclic voltammogramic (CV) curves and Nyquist plots of step-by-step modifications on the electrode surface in phosphate buffer. The results demonstrated that the formation of the monolayer AuNPs could increase the reaction area and decrease the impedance of the electrode.

### 3.2. Characterization of the Extracted Target dsDNA

**Figure 2:**
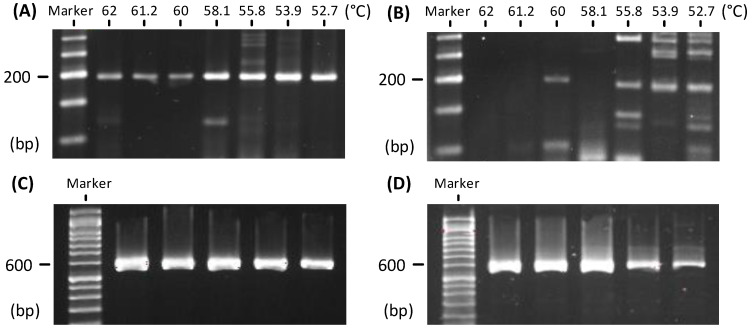
Gel electrophoresis analysis results comparing the hybridization efficiency between the extracted target dsDNA and the P35S primer at various temperatures. (**A**) dsDNA extracted from GMS, (**B**) dsDNA extracted from OS, (**C**) confirmation of the target dsDNAs extracted from GMS containing the soybean gene fragment Rbcl, (**D**) confirmation of the dsDNA extracted from OS containing the soybean gene fragment Rbcl.

In this study, the complementary characteristics of DNA sequence decomposition at high temperature and adhesion at low temperature were used to hybridize the target dsDNAs with the capture ssDNA probes. As different DNAs have their own specific hybridization reaction temperatures, it is necessary to perform a hybridization reaction temperature analysis to optimize the hybridization performance between the target dsDNA and the capture ssDNA probe. Such an analysis also improves the complementary selectivity of the capture ssDNA probe to the target dsDNA. Figure 2 shows the gel electrophoresis analysis results of the hybridization efficiency between the extracted target dsDNA and the P35S primer at various temperatures. Figure 2A,B are the results for the target dsDNA extracted from GMS and OS, respectively. From these results, it can be concluded that the optimal hybridization temperature for both GM and OS soybeans is 61.2 °C. At this temperature, gel electrophoresis did not produce tailing nor amplify the DNA of other base pairs. Because the extracted DNA from OS did not hybridize with P35S primer to generate a PCR amplification reaction, there is no band at the 200 base pair in Figure 2A.

Furthermore, to confirm that the target dsDNAs were extracted from real soybeans, a hybridization of the Rbcl primer of the soybean gene fragment Rbcl and the target dsDNA was conducted at 61.2 °C. The gel electrophoresis analysis results of the DNA extracted from GMS, shown in Figure 2C, indicated that a visible band can be observed at the 600 base pair. This band can also be seen in the analysis results of the DNA extracted from OS shown in Figure 2D. The experimental results comparing the hybridization between the Rbcl primer and the extracted DNA confirmed that the dsDNAs were extracted from real soybeans.

### 3.3. GMS Detection

(1) EIS characteristics of the sensing electrode

**Figure 3:**
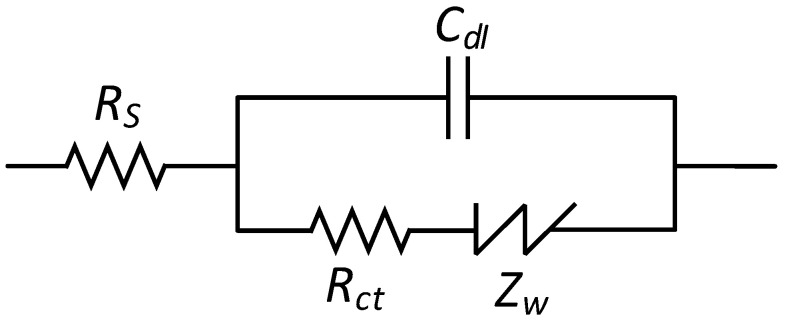
The Randles and Ershler circuit model. *R_S_*: solution resistance; *Z_W_*: diffusion efficiency, *C_dl_*: electric capacity, *R_ct_*: charge transfer resistance on the electrode.

EIS was used for the characterization of the proposed sensing electrode. All experimental results were fitted using the Randles circuit model [34]. This consists of an active electrolyte resistance *R_S_* in series with the parallel combination of the constant phase element (CPE), and a series composed of the charge transfer resistance on the electrode (*R_ct_*) and the diffusion efficiency (*Z_W_*) (Figure 3). Usually, the value of each *R_S_* is much smaller and can be neglected when compared with its corresponding *R_ct_* value. Hence, the Randles’ equivalent circuit can be described as:

(1) $$\begin{array}{ll} Z(\omega ) & =\frac{R_{ct}}{1\;+\;\omega^{2}R_{ct}{}^{2}C_{dl}{}^{2}}-j\frac{\omega R_{ct}{}^{2}C_{dl}}{1\;+\;\omega^{2}R_{ct}{}^{2}C_{dl}{}^{2}}\\ & =R+jX \end{array}$$

As described in Equation (1), both *R_ct_* and *C_dl_* affect the impedance plot. In general, the change in *R_ct_* is more substantial when compared with *C_dl_*. Therefore, the variation of the charge transfer resistance between the target DNA hybridized electrode and the MCH modified electrode (∆*R_ct_*) can be used as the index for GMS detection, as described below.

(2) $$\Delta R_{ct}=R_{ct\;(\mathrm{target}\;\mathrm{DNA})}-R_{ct\;(\mathrm{MCH})}$$

**Figure 4:**
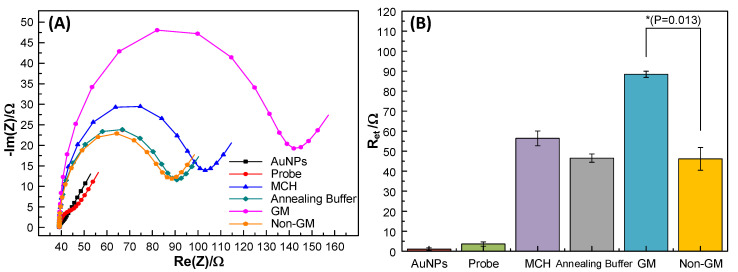
EIS results of each modification step on the electrode. (**A**) Nyquist plots (MCH = 5 mM, Target dsDNA = 20%), (**B**) bar graph of (**A**), N = 6. Student’s t-test statistics: significantly different (*): *p* < 0.05.

Three types of molecules were used as the sensing targets to characterize the sensing electrode. Figure 4 shows the EIS results of each step of the sequential modifications on the electrode. The Nyquist plots illustrated in Figure 4A reveal that the *R_ct_* of the AuNP electrode and the ssDNA probe-immobilized electrode were small. Since DNA is negatively charged, the *R_ct_* of the probe-immobilized electrode was small. Because MCH is an organic compound containing the thiol functional group linked to an alkyl chain, the *R_ct_* of the MCH immobilized electrode increased. However, the *R_ct_* of the annealing buffer-added electrode was smaller than that of the MCH immobilized electrode. This reduction in *R_ct_* could be attributed to the electrolyte ions in the annealing buffer that enhanced the electron transfer in the solution, hence reducing the *R_ct_*. Since the target DNA extracted from the OS was diluted using the annealing buffer and the OS DNA would not hybridize with the capture DNA probe, the *R_ct_* of the OS DNA-added electrode was about the same as that of the annealing buffer-added electrode. The *R_ct_* of the GMS DNA-added electrode further increased due to the successful hybridization between the GMS DNA and the capture DNA probe. The corresponding bar graph for Figure 4A is depicted in Figure 4B. The bar graph indicates that there was a significant R_ct_ difference between the GMS DNA-modified and the OS DNA-modified electrode.

(2) Calibration curve for GMS detection

**Figure 5:**
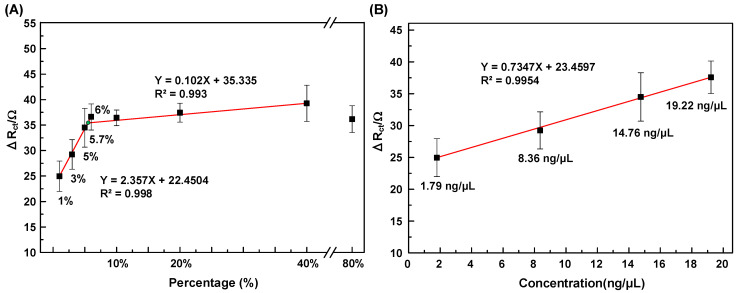
Standard curve for GMS detection. (**A**) Percentage stand curve, (**B**) concentration standard curve.

Different percentages, 1%, 3%, 5%, 5.7%, 6%, 10%, 20%, 40%, and 80% of the extracted GMS DNA were employed for the construction of the calibration curve for GMS detection. Figure 5A shows the percentage while Figure 5B shows the concentration calibration curves for GMS DNA detection. As shown in Figure 5A, two percentage calibration curves were obtained. The calibration curve corresponding to the lower percentage range (1–6%) exhibits a sensitivity of 2.36 Ω/% with R^2^ = 0.9983, while the calibration curve corresponding to the higher percentage range (6–40%) possesses a sensitivity of 0.1 Ω/% with R^2^ = 0.9928.

The Invitrogen Qubit 4 fluorometer and the Qubit™ 1X dsDNA HS Assay Kit were used to convert the percentage of target DNA to concentration. The low percentage standard curve (1%, 3%, 5% and 6%) shown in Figure 5A was converted to the concentration standard curve, as shown in Figure 5B. The sensitivity and R^2^ were 0.735 Ω/(ng/μL) and 0.995, respectively.

Two target DNA samples, 4% and 5.7%, were used to verify the accuracy of the measurement using the concentration standard curve shown in Figure 5B. The results are listed in Table 1, wherein the measured concentration and the real concentration denote the concentration using the concentration standard curve and the Invitrogen Qubit 4 fluorometer, respectively. The recovery rates for the 4% and 5.7% GMS DNA were measured to be 104.1% and 102.49%, respectively. Both are within the acceptable recovery rate range of 80–120% with a good relative standard deviation (RSD). The real sample detection results confirmed that the proposed sensing scheme could precisely measure the GMS DNA.

(3) Performance comparisons

In recent years, many GMS detection devices based on a nanostructured electrode have come under increasing investigation. For comparison, the functional properties of the proposed monolayer AuNPs electrode and those of other recently developed sensing devices are shown in Table 2. Although the linear detection range and LOD of our device are not as effective as those of other reported works, our sensing scheme possesses some advantages. The major advantage is that the monolayer AuNP’s electrode of our device is simple and suitable for mass production and real applications. The aim of this study was to directly detect the GMCs through EIS. Therefore, the standard detection curve of our device was constructed using real GMS DNAs directly extracted from soybeans without PCR amplification or indicator treatment, hence the linear range was different from the published works. The experimental results of this study indicated that the linear detection range of the proposed genosensor could detect the contents of GMCs which can comply with local regulations. It is also hoped that the results of this research can be applied to the qualitative and quantitative testing of specific genetic traits of other GMCs in the future.

## 4. Conclusions

Genetically modified foods have been reported to have an impact on human health. Therefore, GMC is a modern issue of global concern. Compared to the traditional PCR technique, we proposed a low-cost, simple operation, rapid detection genosensor based on an electrode of monolayer AuNPs for the detection of GMS. The proposed sensing scheme enables the direct detection of GMCs using the target DNA extracted from soybeans without additional treatment. The calibration curve corresponding to the lower percentage range (1–6%) exhibits a sensitivity of 2.36 Ω/% with R^2^ = 0.9983, while the calibration curve corresponding to the higher percentage range (6–40%) possesses a sensitivity of 0.1 Ω/% with R^2^ = 0.9928. The recovery rates for the 4% and 5.7% GMS DNA were measured to be 104.1% and 102.49% with RSD at 6.24% and 2.54%. Experimental results demonstrate that our genosensor can successfully detect GMS. It is also hoped that the results of this research can be applied to the qualitative and quantitative testing of specific genetic traits of other GMCs in the future.

## Figures and Tables

**Table 1:** Real sample detection (N = 3).

Con. (%)	Measured Con. (ng/μL)	Real Con. (ng/μL)	Recov. Rate (%)	RSD (%)
4%	11.399	10.96	104.01	6.24
5.7%	16.912	16.5	102.49	2.54

**Table 2:** Performance comparison of recently developed GMS detection biosensors.

Electrode	Detection Method	Sample Type	Linear Range	LOD	Refer.
AuNPs/MWCNT-rGONR	EIS (label-free)	PCR amplify.	1 × 10^−16^–5 × 10^−10^ M (PCR)	3.3 × 10^−17^ M (PCR)	[23]
ROG/CPE	DPV with Methylene blue indicator	PCR amplify.	3 × 10^−15^–5 × 10^−7^ M (PCR)	1.7 × 10^−5^ M (PCR)	[35]
AuNPs-rGO	PEC with (SiO_2_@CdTe QDs) indicator	PCR amplify.	1 × 10^−16^–5 × 10^−10^ M (PCR)	5 × 10^−17^ M (PCR)	[36]
Poly(nBA-NAS)-rGO	DPV with (AQMS) indicator	Real sample	1 × 10^−15^–1 × 10^−8^ M (Synthetic)	6.3 × 10^−16^ M (Synthetic)	[37]
Monolayer AuNPs	EIS (label-free)	Real sample	1.792–1.922 × 10^1^ ng/mL	1.792 ng/mL	This work

## Data Availability

Not applicable.
